# Ineffectiveness of the 2014-2015 H3N2 influenza vaccine

**DOI:** 10.18632/oncotarget.6746

**Published:** 2015-12-23

**Authors:** Michal Mandelboim, Aharona Glatman-Freedman, Yaron Drori, Hilda Sherbany, Rakefet Pando, Hanna Sefty, Hila Zadka, Tamar Shohat, Nathan Keller, Ella Mendelson

**Affiliations:** ^1^ Central Virology Laboratory, Ministry of Health, Chaim Sheba Medical Center, Ramat-Gan, Israel; ^2^ The Israel Center for Disease Control, Israel Ministry of Health, Tel-Hashomer, Israel; ^3^ Departments of Pediatrics and Family and Community Medicine, Valhalla, New York, USA; ^4^ Department of Epidemiology and Preventive Medicine, School of Public Health, Sackler Faculty of Medicine, Tel-Aviv University, Tel-Aviv, Israel; ^5^ Department of Clinical Microbiology, Sheba Medical Center, Tel-Hashomer, Israel; ^6^ Ariel University Centre, Ariel, Israel

**Keywords:** influenza, vaccine, Immunology and Microbiology Section, Immune response, Immunity

## Abstract

The seasonal influenza vaccine is currently the most effective preventive modality against influenza infection. Nasopharyngeal samples of vaccinated and non-vaccinated patients presenting with Influenza-like-illness (ILI) were collected from over 20 outpatient clinics located in different geographic parts of Israel and were tested for the presence of influenza viruses (influenza A and influenza B). Here we show, that in the 2014-2015 season, the vaccine that included the A/Texas/50/2012 H3N2 virus was ineffective. Significant numbers of individuals vaccinated with the 2014-2015 vaccine, of all ages, were infected with influenza A (H3N2), manifesting similar symptoms as the non-vaccinated group. We further demonstrate that the Israeli circulating influenza A(H3N2) virus was different than that included in the 2014-2015 northern hemisphere vaccine, and that antibodies elicited by this vaccine were significantly less efficient in neutralizing influenza A(H3N2) infection.

## INTRODUCTION

Influenza virus is a major cause of morbidity and mortality around the world [1, 2]. In addition, influenza infections are a source of a great economic burden [3]. It is a single strand RNA virus that encodes for 13 genes among them are the neuraminidase and the hemagglutinin proteins that are expressed on the virus itself and on the surface of the infected cells. The virus is subjected to rapid and significant changes that prevents the generation of a long-lasting protective immunity [4, 5]. Indeed, around 10,000 different sequences of the hemagglutinin and the neuraminidase proteins are found in data banks. Thus, a yearly vaccine that includes 3 to 4 influenza virus strains, the identity of which is decided annually based on the circulating influenza viruses, is the primary preventive strategy against the influenza virus [6]. During the 2014-2015 influenza season, it has become evident that multiple influenza A(H3N2) virus isolates from the north hemisphere do not match with the influenza A(H3N2) included in this season's vaccine strain (CDC reports [7] and [8]). Here we examined the effect of the north hemisphere 2014-15 influenza vaccine that includes the influenza A(H3N2) A/Texas/50/2012 virus on the drifted virus isolated in Israel.

## RESULTS

### Infection of vaccinated individuals with Influenza A (H3N2)

**Figure 1:**
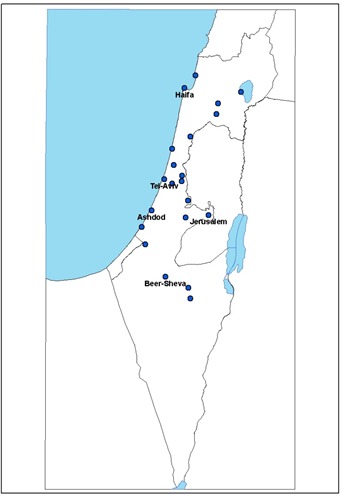
Location of the clinics in different geographic parts in Israel

Nasopharyngeal samples of patients presenting with Influenza-like-illness (ILI) were collected from over 20 outpatient clinics located in different geographic parts of Israel (Figure 1) and tested for the presence of influenza viruses (influenza A and influenza B). From the 40th week of 2014 until the 10th week of 2015, 1048 samples were collected, of which 309 (27.5%) were positive for influenza; of these 269 (87%) were positive for influenza A(H3N2) virus, 15 (4.85%) for influenza A(H1N1)pdm09, 4 (1.29%) were un-subtyped influenza A and 19 (6.14%) were infected with influenza B virus. The relatively large proportion of cases infected with H3N2 virus prompted us to investigate the circulating A(H3N2) influenza virus in Israel.

**Figure 2:**
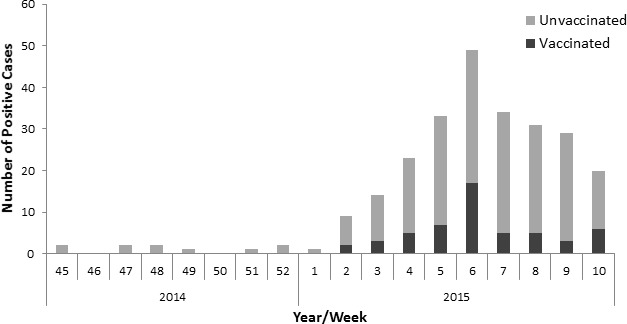
Vaccination status of patients infected with H3N2 influenza The graph demonstrates the number of influenza A (H3N2)-positive patients and their vaccination status.

**Figure 3:**
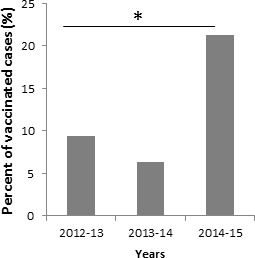
Percentages of vaccinated and infected individuals from 2012-2015

All ILI patients that were positive for influenza A(H3N2) prior to week 49 of 2014 were not immunized. However, starting from the 2nd week of 2015 we observed that relatively large proportion (54 out of 254, 21.25%) of the patients infected with influenza A(H3N2) influenza were vaccinated at least one time (Figure 2). To determine whether the percentages of people who were vaccinated against influenza and still infected was indeed increased in 2014-2015 we compared the percentages of vaccinated and infected people in 2014-2015 to previous years. As can be seen, significantly more vaccinated people were infected with influenza as compared to previous two years 2012-2013 and 2013-2014 (Figure 3).

### The circulating influenza A(H3N2) strains belong to the 3C.2a group, while the vaccine strain is part of the 3C.1 group

To determine whether the influenza A(H3N2) circulating in Israel differs from the 2014-2015 influenza A(H3N2) vaccine strain, we isolated viruses from 22, randomly selected, 9 vaccinated and 13 non-vaccinated individuals and performed molecular characterization.

**Figure 4:**
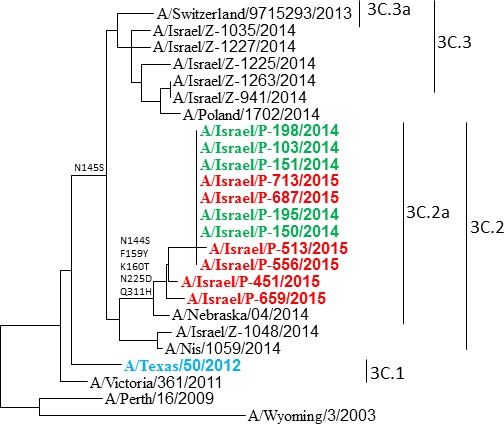
Phylogenetic analysis of influenza A (H3N2) isolated in Israel during the 2014-2015 season Phylogenetic tree generated by comparing the 928 nucleotides of the HA protein. The northern hemisphere 2014-2015 vaccine strain A/Texas/50/2012 is shown in light blue. Strains isolated from vaccinated individuals are shown in red and from non-vaccinated individuals in green. Influenza A(H3N2) isolated during the 2013-2014 season and reference strains are shown in black.

As seen in the phylogenetic analysis (Figure 4), the influenza A(H3N2) that circulated in Israel differs from the influenza A(H3N2) A/Texas/50/2012 strain found in the 2014-2015 northern hemisphere vaccine, and from the influenza A(H3N2) strains that were detected in Israel during the 2013-2014 season. While the vaccine influenza A(H3N2) strain belongs to the 3C.1 group, the strains isolated in Israel belong to the 3C.2 and 3C.3 group, and those isolated in 2015 belong to the to the 3C.2a group. The amino acid differences are indicated in Table 1. No difference was observed between the 2014-2015 influenza A(H3N2) strains isolated from vaccinated and non-vaccinated individuals (Figure 4).

**Table 1:** Comparison of the amino acid differences between the vaccinated strain, the Israeli circulating strain and the vaccinating strain of the coming year

Amino Acid	A/Texas/50/2012		A/Israel/P-151/2014		A/Switzerland/9715293/2013	
3	CTT	L	ATT	I	CTT	L
128	AAT	N	ACT	T	GCT	A
138	GCT	A	GCT	A	TCT	S
142	AGA	R	AGA	R	GGA	G
144	AAT	N	AGT	S	AAT	N
145	AAT	N	AGT	S	AGT	S
159	TTC	F	TAC	Y	TCC	S
160	AAA	K	ACA	T	AAA	K
186	GTT	V	GGT	G	GGT	G
198	CCA	P	TCA	S	TCA	S
219	TTT	F	TCT	S	TCT	S
225	AAT	N	GAT	D	GAT	D
311	CAA	Q	CAT	H	CAA	Q
326	AAA	K	AAA	K	AGA	R
489	GAT	D	AAT	N	GAT	D

### Clinical presentation of vaccinated and unvaccinated influenza-positive individuals

**Figure 5:**
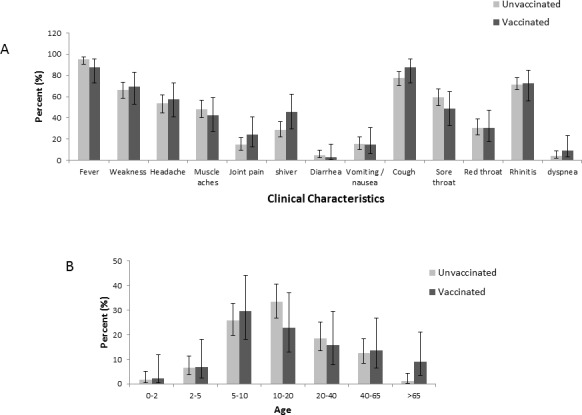
Symptoms and ages The figure demonstrates the symptoms and signs (A) and age (B) of patients infected with influenza A(H3N2) virus. The total number of patients in each group (vaccinated and non-vaccinated) were set to be 100%.

Most of the patients suffered from fever, cough, rhinitis and weakness (Figure 5A and Supplementary Table 1) and no statistically significant differences were observed in the symptoms and signs of vaccinated and the non-vaccinated influenza A(H3N2) individuals evaluated (Figure 5A and Supplementary Table 1). In addition, no significant differences were observed between the ages of vaccinated and the non-vaccinated influenza A(H3N2) individuals (Figure 5B and Supplementary Table 2).

### Antibody response to the circulating influenza A(H3N2) in individuals vaccinated with the 2014-2015 northern hemisphere vaccine

**Figure 6:**
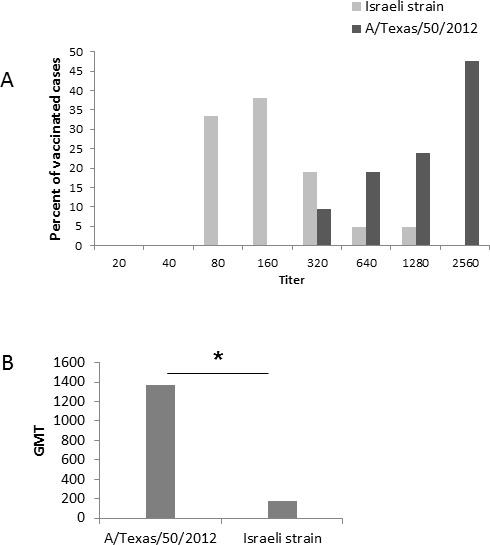
Microneutralization assay using serum of individuals vaccinated with the northern hemisphere 2014-2015 influenza vaccine **A.** Antibody titers against the Israeli circulating influenza A(H3N2) strain (A/Israel/P-151/2014) and the vaccine influenza strain (A/Texas/50/2012). **B.** Geometric Mean Titers (GMT) antibody neutralization of the Israeli circulating influenza A(H3N2) and the vaccine influenza A(H3N2) strain. *P < 0.0001 using the chi-square test.

To determine the ability of antibodies elicited by the 2014-2015 influenza vaccine, to neutralize the new circulating influenza A(H3N2) virus, we obtained sera from 21 individuals, that had received the 2014-2015 influenza vaccine and tested them against the influenza A(H3N2) A/Texas/50/2012 and the influenza A(H3N2) A/Israel/P-151/2014 virus using a microneutralization assay [9]. Positive controls were for the A/Texas/50/2012 antibodies that were obtained from the WHO and gave a titer of 2560. For the Israeli strains we obtained sera from confirmed-infected individual that had a titer of 2560. As can be seen in Figure 6A, immunization with the A/Texas/50/2012 virus resulted in the generation of antibodies that reacted with both strains. However, higher dilutions of the sera were better able to block the infection of the influenza A(H3N2) A/Texas/50/2012 virus as compared to the Israeli influenza A(H3N2) A/Israel/P-151/2014 virus. Consequently, the average Geometric Mean Titers (GMT) against A/Texas/50/2012 was significantly higher than the Israeli influenza A(H3N2) A/Israel/P-151/2014 virus (Figure 6B).

## DISCUSSION

We show here that relatively large proportion of individuals vaccinated with the 2014-2015 northern hemisphere vaccine were infected with the influenza A(H3N2) Israeli strain. We further demonstrated that antibodies elicited by vaccination had reduced ability to neutralize the influenza (H3N2) circulating in Israel; the diseases presentation was similar in ILI individuals infected with influenza A(H3N2) irrespective of whether they were vaccinated or not.

Phylogenetically we show that the influenza A(H3N2) viruses obtained from vaccinated and non-vaccinated individuals are different than the vaccinating strain. The Israeli viruses isolated from all individuals belong to the 3C.2a, while the vaccinating strain A/Texas/50/2012 virus belongs to the 3C.1 group. Similar 3C.2a viruses were observed countries in Europe and in the US [7, 10-12]. The hemagglutinin protein of the 3C.2a group is different from that of 3C.1 in several major amino acids that determine the antigenicity of these viruses [10]. This is consistent with our observation that antibodies present in the sera of vaccinated individuals were significantly less efficient in neutralizing the Israeli influenza A(H3N2) infection. We previously reported that naturally occurring antibodies cross react with several influenza viruses [13]. Some antibody cross-reactivity was found between the Israeli influenza A(H3N2) virus and the A/Texas/50/2012 virus, however it may have been insufficient in modifying the course of infection with the circulating novel influenza A(H3N2) in Israel. Finally, we also show that the circulating Israeli strain is substantially different than the vaccinating strain that is giving to protect infection in the coming year (Table 1). Thus, we predict that also in 2015-2016 the influenza vaccine will be less efficient.

## MATERIALS AND METHODS

### Ethical approval

The institutional review board (IRB) of the Sheba Medical Center approved this research (Helsinki Number 1967-15-SMC). Informed consent was signed by all participants of this study who donated sera.

### Sample collection

Nasopharyngeal samples of patients presenting with Influenza-like-illness (ILI) were collected from over 20 outpatient clinics located in different geographic parts of Israel and are tested for the presence of influenza viruses (influenza A and influenza B). The survey was anonymous and participants were recorded by serial numbers

### Cells

(MDCK) cells were obtained from the WHO collaborating Centre for influenza virus, the national institute for medical research (MRC), London UK. The cells were used for a maximum of 25 passages and maintained in Dulbecco's modified Eagle's medium (Biological Industries, Israel) containing 10% fetal bovine serum (Biological Industries, Israel), 2 mM l-glutamine, and the antibiotics penicillin and streptomycin.

### Viral RNA extraction and PCR

The extraction of viral RNA was performed with KingFisher (Thermo Electron Corporation, Waltham, MA, USA)

Real-time reverse transcription-PCR (rRTPCR) was performed to determine the Influenza. Reactions were performed the Ambion Ag-Path master mix (Life Technologies, USA) using TaqMan Chemistry on the ABI 7500 instrument

H3 forward primer 5′-AAT GGT TGG GAG GGA ATG-3′, H3 reverse primer 5′-TTG AGT GCT TTT RAG ATC TGC-3′, H3 probe 5′-VIC-TTG GTA CGG TTT CAG GCA TCA-TAMRA-3′, where TAMRA is 6-carboxytetramethylrhodamine.

### Phylogenetic trees

To establish a phylogenetic tree, from randomly selected samples. The specific primers used were:

H3N2-F6 H3N2-F6 5′ AAGCAGGGGATAATTCTATTAACC 3′

H3HA-R1075 5′ AACCGTACCAACCRTCCACCATTC 3′

RT-PCR products were sequenced using ABI PRISM Dye Deoxy Terminator cycle sequencing kit (Applied Biosystems, Foster City, CA). Reaction mixtures were analyzed on Applied Biosystems

The Sequencher® 5.0 program (Gencodes Corporation, Ann Arbor, MI) was used to compare the nucleotide sequences. Phylogenetic trees were prepared by nearestneighbor joining analysis using Clustal X with 1000 bootstraps and trees were visualized using TreeView or NJ plot software.

Selected sequences were deposit in GenBank (KT006534-KT006349).

### Microneutralization assay

Human sera were heat inactivated, and two fold serial dilutions of 1:20-1:2560 were performed in immunoassay plates. The diluted sera were mixed with an equal volume of the same medium containing 100 TCID50/50 ml of influenza virus (A/Texas/50/2012 or the Israeli strain A/Israel/P-151/2014). After 1 hour incubation at 37° MDCK cells were added to each well. The plates were incubated for 20 hour at 37°C. The supernatant was removed, and monolayers were washed with PBS and fixed in 80% cold acetone for 10 minutes.

### ELISA assays

The presence of viral protein was detected by ELISA as previously described [9, 14]. Specifically, the presence of viral protein was detected by ELISA with a monoclonal antibody to the influenza A NP (clone A-1 and A-3, Millipore). The fixed plates were washed three times with PBS containing 0.05% Tween 20 (wash buffer). The anti-NP antibody diluted 1/4,000 in PBS containing 1% bovine serum albumin and 0.1% Tween 20 (E diluent) was added to each well. The plates were incubated at room temperature for 1 h. The plates were washed four times in wash buffer, and 100 μl of horseradish peroxidase-labeled goat anti-mouse immunoglobulin G (IgG) (Dako, USA) diluted 1/2,000 in E diluent was added to each well. The plates were incubated for 1 h at room temperature and then washed six times with wash buffer. One hundred microliters of freshly prepared substrate (10 mg of o-phenylenediamine dihydrochloride per 20 ml of 0.05 M phosphate citrate buffer, pH 5.0, containing 0.03% sodium perborate) was added to each well, and the plates were incubated at room temperature for approximately 5 min. The reaction was stopped with an equal volume of 1 N sulfuric acid. The absorbance was measured at 490 nm (A490).

### Statistical analysis

A Binomial 95% confidence interval was calculated for this rate. The chi-square test was applied to evaluate the differences. A p value <0.05 was considered to be statistically significant. All analyses were performed using SPSS (version 21.0.0. SPSS Inc., Chicago, IL, USA), SAS (SAS 9.1, SAS Institute Inc, Cary, NC, USA) and Excel softwares.

## SUPPLEMENTARY MATERIAL TABLES


