# Supplementary material for: Ineffectiveness of the 2014-2015 H3N2 influenza vaccine
Source: Oncotarget. 2015 Dec 23;7(2):1185–92. doi: 10.18632/oncotarget.6746 (PMC4811452; doi:10.18632/oncotarget.6746)
Supplement: Supplementary file 1 [file oncotarget-07-1185-s001.pdf]

Ineffectiveness of the 2014-2015 H3N2 influenza vaccine

Supplementary Material

Supplementary Table 1: data related to figure 3A

Symptoms	Vaccinated		Unvaccinated	
	Number (n=33)	Percent (95%CI)	Number (n=143)	Percent (95%CI)
Fever	29	87.88 (72.67-95.18)	136	95.1(90.24-97.61)
Weakness	23	69.7 (52.66- 82.62)	95	66.43 (58.35-73.65)
Headache	19	57.58 (40.81-72.76)	77	53.85 (44.68-61.81)
Muscle aches	14	42.42 (27.24-59.19)	69	48.25 (40.22-56.38)
Joint pain	8	24.24 (12.83-41.02)	21	14.69 (9.81-21.41)
shiver	15	45.45 (29.84-62.01)	41	28.67 (21.89-36.56)
Diarrhea	1	3.03 (0.54-15.32)	7	4.9 (2.39-9.76)
Vomiting / nausea	5	15.1 (6.65-30.92)	22	15.36 (10.38-22.2)
Cough	29	87.88 (72.67-95.18)	111	77.62 (70.12-83.68)
Sore throat	16	48.48 (32.5-64.78)	85	59.44 (51.25-67.14)
Red throat	10	30.3 (17.38-47.34)	44	30.77 (23.79-38.75)
Rhinitis	24	72.73 (55.78-84.93)	102	71.33 (66.44-78.11)
dyspnea	3	9.09 (3.14-23.17)	6	4.2 (1.94-8.85)

Supplementary Table 2: Data for Figure 3B

Age	Vaccinated		Unvaccinated	
	Number (n=44)	Percent (95%CI)	Number (n=167)	Percent (95%CI)
**0-2**	1	2.27 (0.4-11.81)	3	1.8 (0.61-5.15)
**2-5**	3	6.82 (2.35-18.23)	11	6.59 (3.72-11.41)
**5-10**	13	29.55 (18.16-44.22)	43	25.75 (19.71-32.87)
**10-20**	10	22.73 (12.84-36.99)	56	33.53 (26.81-40.49)
**20-40**	7	15.91 (7.93-29.37)	31	18.56 (13.4-25.14)
**40-65**	6	13.64 (6.4-26.71)	21	12.57 (8.37-18.46)
**>65**	4	9.09 (3.59-21.16)	2	1.2 (0.33-4.26)
